# Biophysical and structural studies of fibulin-2

**DOI:** 10.1038/s41598-024-64931-7

**Published:** 2024-07-02

**Authors:** Anil A. Sohail, M. Kristian Koski, Lloyd W. Ruddock

**Affiliations:** 1 Faculty of Biochemistry and Molecular Medicine, University of Oulu, 90220 Oulu, Finland; 2 Biocenter Oulu, University of Oulu, 90220 Oulu, Finland

**Keywords:** Biochemistry, Cell biology, Structural biology

## Abstract

Fibulin-2 is a multidomain, disulfide-rich, homodimeric protein which belongs to a broader extracellular matrix family. It plays an important role in the development of elastic fiber structures. Malfunction of fibulin due to mutation or poor expression can result in a variety of diseases including synpolydactyly, limb abnormalities, eye disorders leading to blindness, cardiovascular diseases and cancer. Traditionally, fibulins have either been produced in mammalian cell systems or were isolated from the extracellular matrix, a procedure that results in poor availability for structural and functional studies. Here, we produced seven fibulin-2 constructs covering 62% of the mature protein (749 out of 1195 residues) using a prokaryotic expression system. Biophysical studies confirm that the purified constructs are folded and that the presence of disulfide bonds within the constructs makes them extremely thermostable. In addition, we solved the first crystal structure for any fibulin isoform, a structure corresponding to the previously suggested three motifs related to anaphylatoxin. The structure reveals that the three anaphylatoxins moieties form a single-domain structure.

## Introduction

Fibulin-2 is one of eight members of the fibulin family found in mammals^1^. Fibulin-1, the first member of the fibulin family, was distinguished from other basement membrane proteins by having two distinct cysteine rich regions^2^. The first region has close homology to anaphylatoxin-like modules, while the second has homology to epidermal growth factor (EGF)-like domains^3^. Fibulin-2 was subsequently recognized as a member of the fibulin family^4,5^ and shows approximately 45% sequence identity with fibulin-1^6–9^. Fibulin-2 also contains a unique N-domain, which shows no sequence similarity with other extracellular matrix (ECM) proteins.

**Figure 1:**
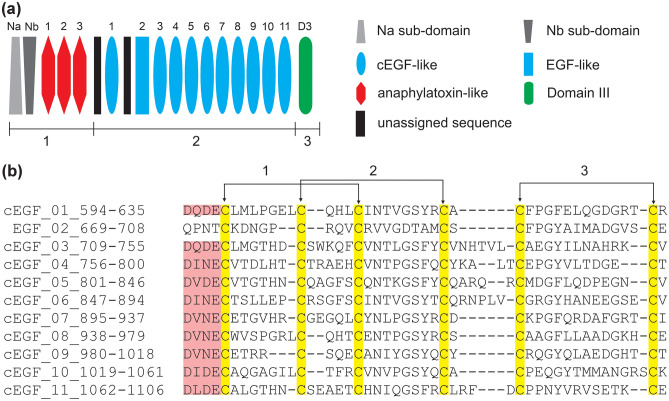
Primary structure of fibulin-2. (**a**) A schematic representation of mature mouse fibulin-2. It is divided into three regions, 1–3. Domain representations are not drawn to scale. (**b**) Sequence alignment of the ten calcium binding cEGF-like domains and one non-calcium binding EGF-like domain of region 2. Cysteines are highlighted in yellow and the disulfide bond pattern between the cysteines are shown by arrows. The calcium binding residues are highlighted in pink.

Mouse fibulin-2 has 83.7% identity with the human protein and can be divided into three regions 1–3 (Fig. 1a)^4,5^. The N-domain (V27-T434) can be further divided into sub-domains comprised of N_a_ (V27-C176) and N_b_ (H177-T434). N_a_ is a cysteine rich sub-domain having 22 cysteines whereas the N_b_ sub-domain lacks cysteines. The three anaphylatoxin-like modules (C435-C543) following the N-domain are also found in fibulin-1, but not in other fibulins. These modules have a total of 17 cysteines which are predicted to form 8 disulfide bonds along with an unpaired cysteine (C500)^4,10^. Region 2 of fibulin-2 is comprised of eleven EGF-like domains having in total 66 cysteines, with each domain having 6 conserved cysteine residues (Fig. 1b)^4^. Two unassigned sequences (E544-D593 and P636-P668) are found before and after the first EGF-like domain (D594-R635). The third EGF-like domain (D709-V755) in the mouse protein is absent in human fibulin-2. Ten of the EGF-like domains of mouse fibulin-2 are reported to be calcium binding domains (cEGF) having a consensus sequence D-x-D/N-E before the first cysteine (highlighted pink in Fig. 1b)^11^. These residues are not found in the second EGF-like domain (Q669-E708). The C-terminal region known as Domain III (R1107-P1221) has two cysteines and is ubiquitous in the fibulin family^12^.

According to electron microscopy studies, the overall shape and size of full-length fibulin-2 is different from that of other fibulins^13^. This could be due to the presence of the unique N-domain of region 1 and/or it being the only homodimer fibulin isoform. It has been reported that the free unpaired cysteine residue (C500) present in the region 1 in the second anaphylatoxin-like module plays a role in stabilizing anti-parallel homodimer formation^10^. Additionally, non-covalent interactions between the N-domain and the region 2 EGF-like domains also assist in dimerization^4,10^.

Complex formation between fibulin-2 and other ECM proteins including aggregan, versican, brevican^14^, fibrillin-1^15^, laminins^16,17^, nidogens^18^, perlecan^19^ and tropoelastin^20,21^, have been reported. These complexes play important roles in many biological processes. Fibulin expression is found during the development of elastic fiber structures such as cartilages, cardiac valves, and blood vessels^8^. Malfunction or low fibulin expression may result in several pathological processes, including synpolydactyly and limb abnormalities^22^, eye disorders leading towards blindness^23^, cardiovascular diseases and cancer^24^.

Currently, there are no crystal structures reported for any fibulin family members, but there is an unpublished nuclear magnetic resonance (NMR) structure for the EGF-like 1 domain of human fibulin-4 (PDB code 2KL7). This lack of structural information may arise due to the high level of post-translational modifications in the protein, in particular the high disulfide density. To our knowledge, here we report for the first time the soluble production of mouse fibulin-2 constructs using an *Escherichia coli* (*E. coli*) production system. The constructs made covered 62% of the mature protein (749 out of 1195 residues) and include 42 of the predicted 53 disulfide bonds in the full-length protein. Purified constructs were subjected to biophysical characterization. The first crystal structure within the fibulin family, of the three anaphylatoxin-like modules of mouse fibulin-2, was solved.

## Results and discussion

### Construction and production

Previously, we have successfully produced large and complex disulfide-bond containing ECM proteins using CyDisCo (cytoplasmic disulfide bond formation in *E. coli*) technology^25^. This encouraged us to attempt to produce full-length mature fibulin-2 (V27-P1221). Unfortunately, the soluble production of the full-length construct was not successful in this system and therefore smaller constructs were made (Table 1), to try to identify the limiting factor(s) for their production.

**Table 1:** Plasmids expressing constructs of mouse fibulin-2 used in this study.

Domain boundaries	Plasmids	Details
V27-G545	pAS85	N-domain and three anaphylatoxin-like modules
V27-D593	pAS87	N-domain and three anaphylatoxin-like modules with extension
V27-P1221	pAS86	Full-length mature fibulin-2 protein
S427-G545*	pAS79	Three anaphylatoxin-like modules (wild-type)
S427-G545*	pAS84	Three anaphylatoxin-like modules (mutant C500L)
L590-P1221	pAS88	Eleven EGF-like domains and Domain III
G592-Q710*	pAS71	EGF-like domains; 1 and 2, and unassigned sequence
D709-V802*	pAS69	EGF-like domains; 3 and 4
T800-V896*	pAS70	EGF-like domains; 5 and 6
V894-V981*	pAS75	EGF-like domains; 7 and 8
E979-L1063*	pAS76	EGF-like domains; 9 and 10
K1061-P1221*	pAS77	EGF-like domain 11 and Domain III

Numbering is according to the full-length protein. Fibulin-2 constructs which were solubly produced, purified and characterized are marked (*).

All constructs which included the N-domain of fibulin-2 (V27-G545, V27-D593 and V27-P1221) did not make soluble protein. AlphaFold prediction^26,27^ suggests that the N_b_ sub-domain (H177-T434) of fibulin-2 is unstructured and disordered (supplementary Fig. S1a). From this we hypothesize that this region may interact with some other ECM protein(s) and that such an interaction may be required to stabilize the structure of the N-domain and hence allow soluble production. Constructs containing EGF-like domains were partially solubly expressed, resulting in purified yields in the range 0.1–1.0 mg/L. These production levels are significantly lower than the levels we have observed for other EGF-like containing ECM proteins such as region 3 of perlecan^25^. These differences in protein yield support the idea that protein expression in *E. coli* may be highly dependent on the exact nature of protein of interest and/or dependent on the nature of inter-domain packing in the native protein. In contrast to the low yields of other constructs, the fibulin-2 construct S427-G545 (wild-type and C500L mutant), which has three anaphylatoxin-like modules, was fully solubly produced and was purified in good yields (> 10 mg/L). This construct is predicted to be a disulfide linked homodimer^10^ with a molecular weight of ~ 27.6 kDa and a total of 34 cysteines forming 17 disulfide bonds. Overall, the seven constructs (shown in Table 1) that could be produced as (partly or wholly) soluble proteins covered 62% of fibulin-2, and included 79.3% of the total disulfide bonds.

### Biophysical studies

**Figure 2:**
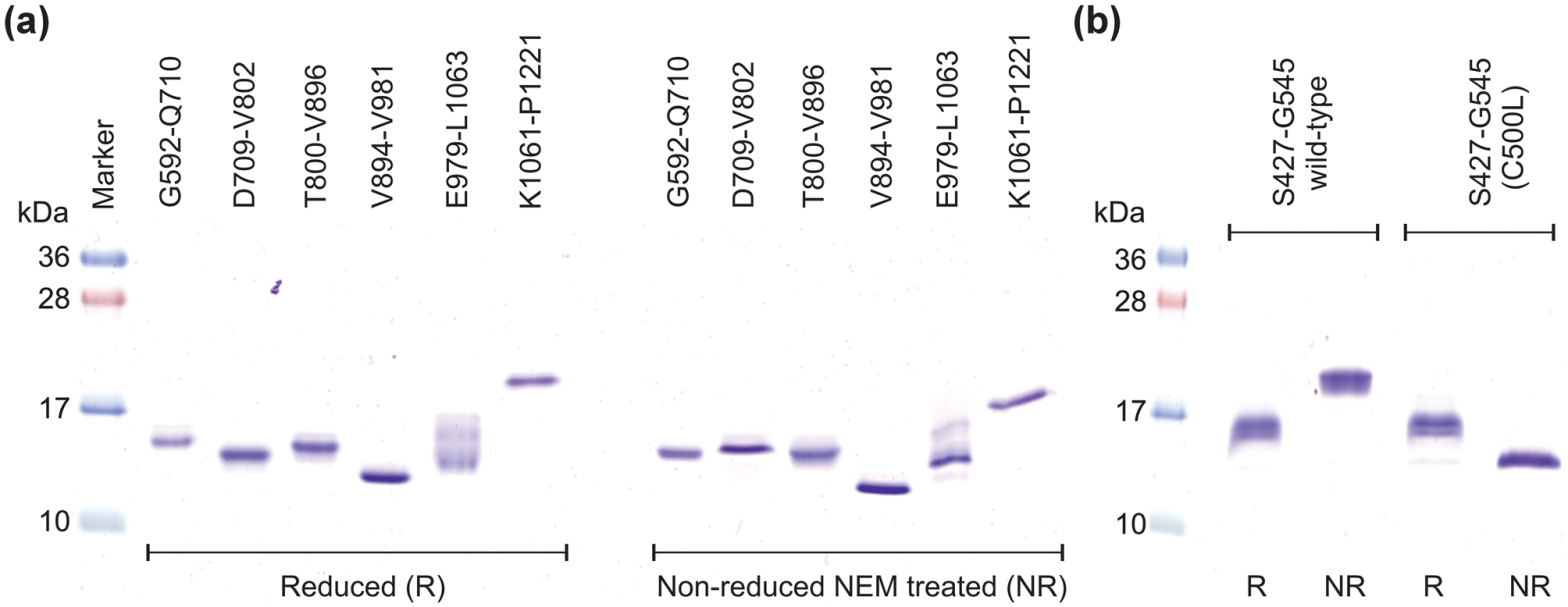
Molecular weight estimation using coomassie stained 15% SDS-PAGE gel of purified fibulin-2 constructs: (**a**) EGF-like domains and (**b**) anaphylatoxin-like modules both wild-type and the C500L mutant.

The apparent molecular weights of all the fibulin-2 purified constructs were analyzed using sodium dodecyl sulfate—polyacrylamide gel electrophoresis (SDS-PAGE). Both the reduced and N-ethylmaleimide (NEM) treated non-reduced purified samples were run on 15% SDS-PAGE gels (Fig. 2). All the EGF-like domain containing constructs, except for E979-L1063, showed a single band for both reduced and non-reduced conditions (Fig. 2a). The presence of multiple bands for E979-L1063 suggests that the construct is prone to degradation and/or modification upon storage in SDS loading buffer as samples run immediately after purification showed a single band. All the bands (except the degradation products of E979-L1063) ran at their expected molecular size in reducing SDS-PAGE and the single band observed in the non-reduced NEM treated samples implies that the constructs all have a single redox state. For the anaphylatoxin-like modules containing construct (S427-G545), a single band near ~ 16 kDa could be seen for the wild-type protein in the reduced state, with a shift in mobility in non-reduced SDS-PAGE indicative of inter-molecular disulfide-based dimerization (Fig. 2b). The apparent molecular weight for the dimer is not twice that of the monomer, probably due to the predicted intramolecular disulfides in each subunit. To confirm this, the C500L mutation was made. This ran at the same position as the wild-type protein in reducing SDS-PAGE, but at a lower molecular weight in non-reducing SDS-PAGE (Fig. 2b). This is consistent with it lacking an intermolecular disulfide which keeps the protein as a homodimer in SDS-PAGE, while retaining intra-molecular disulfides. This implies the C500L mutation disrupts the formation of the inter-molecular disulfide bond in the dimer and that the wild-type protein is all in a disulfide linked homodimer state.

To validate the proteins made and to examine their redox states, the determination of exact molecular weight of the fibulin-2 constructs was done by mass spectrometry (MS) (Table 2). The MS results confirmed that the purified fibulin-2 constructs have the expected molecular weight with all the cysteines present in the constructs being in disulfide bonds. No significant protein adducts having an additional 125 Da molecular weight (or multiple thereof) was seen for any of the NEM-treated samples. This further implies that all the cysteines in the constructs are involved in disulfide bonds. The molecular weight of the C500L mutant and wild-type of the fibulin-2 construct containing three anaphylatoxin-like modules (S427-G545) indicated the monomeric and disulfide-linked dimeric states, respectively.

**Table 2:** Molecular weight analysis by mass spectrometry for purified fibulin-2 constructs.

Construct	No. of cys	Disulfide bonds	MW_theor_ (Da)	MW_exp_ (Da)	MW_Δ_ (Da)
MH_6_M-S427-G545 (monomer)	17	–	13,816	–	–
MH_6_M-S427-G545 (dimer)	34	17	27,632	27,598	34
MH_6_M-S427-G545 (C500L)	16	8	13,826	13,810	16
MH_6_M-G592-Q710	12	6	13,827	13,815	12
MH_6_M-D709-V802	12	6	11,575	11,563	12
MH_6_M-T800-V896	12	6	11,660	11,649	12
MH_6_M-V894-V981	12	6	10,710	10,697	12
MH_6_M-E979-L1063	12	6	10,515	10,503	12
MH_6_M-K1061-P1221	8	4	19,254	19,246	8

The theoretical average molecular weight (MW_theor_) was calculated using ExPASy ProtParam tool ^44^ and compared with the experimentally determined average molecular weight (MW_exp_) of the purified NEM treated fibulin-2 constructs. A reduction of 2 daltons (Da) in the MW_theor_ is expected for the formation of each disulfide bond. MW_Δ_ is the difference between MW_theor_ and MW_exp_.

SEC-MALS analysis was then done, to further investigate the oligomeric states of the S427-G545 constructs (wild-type and C500L mutant). This analysis showed that both the wild-type and C500L mutant eluted in the same volume in SEC. They had an apparent molecular weight of 26.3 kDa and 25.7 kDa (wild-type and C500L mutant, respectively) according to MALS, indicating a dimeric state of both proteins (supplementary Fig. S2). This is in agreement with the study by Sasaki and co-workers^10^, suggesting that the inter-molecular disulfide bond via C500 is not critical for dimer formation.

**Figure 3:**
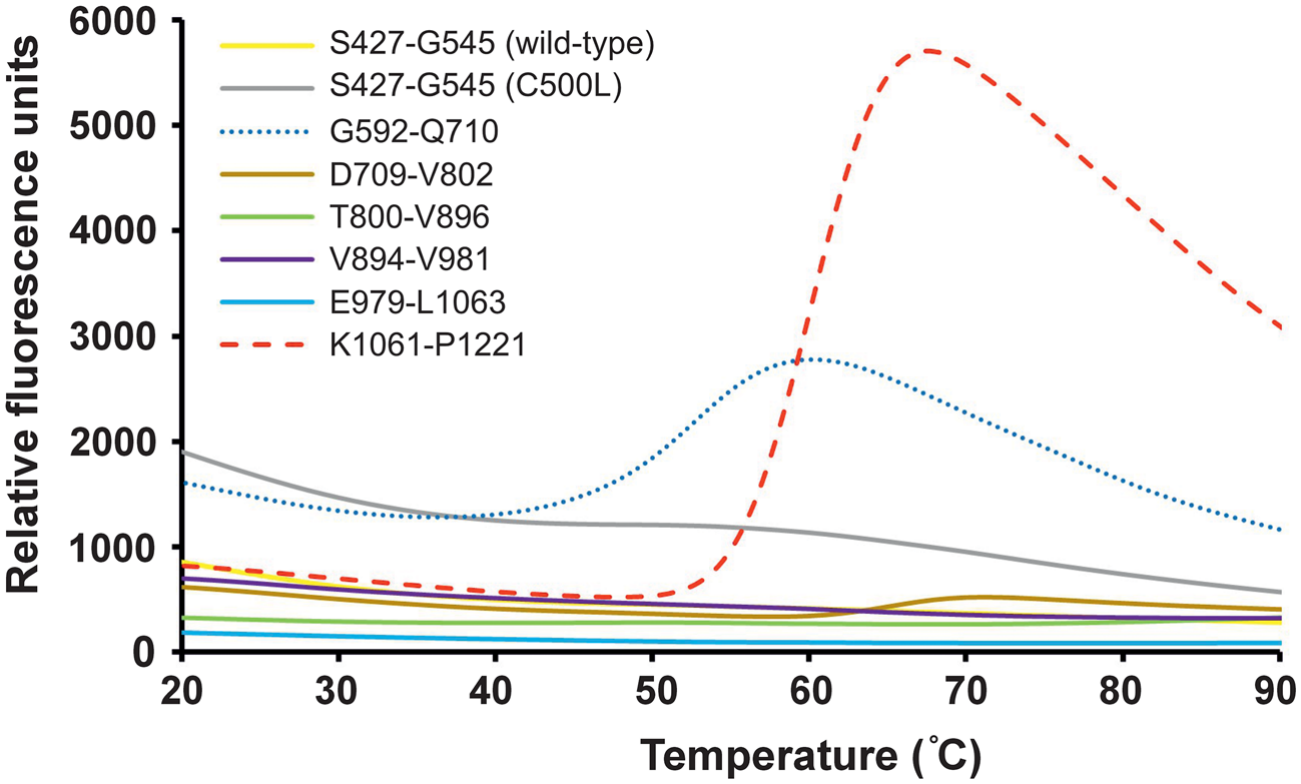
Thermal stability of purified fibulin-2 constructs. The plot shows the change in fluorescence (y-axis) vs temperature (x-axis).

Thermal stability was then examined for all constructs (Fig. 3). There was insignificant change in signal over the temperature range 20–90 ˚C for all of the constructs except for G592-Q710 and K1061-P1221. This suggests the constructs are extremely thermostable, which is consistent with them having multiple disulfide bonds (Table 2). The lower thermal transition shift observed for G592-Q710, could be due to the presence of an unstructured region between the two EGF folds (supplementary Fig. S1c). The thermal stability for the K1061-P1221 construct is relatively lower than other constructs having a single thermal transition shift at 60 ˚C. This could be due to the presence of Domain III, which comprises of 70% of the construct. Domain III has only a single disulfide bond (C1110-C1116) and hence might be expected to be less thermally stable.

**Figure 4:**
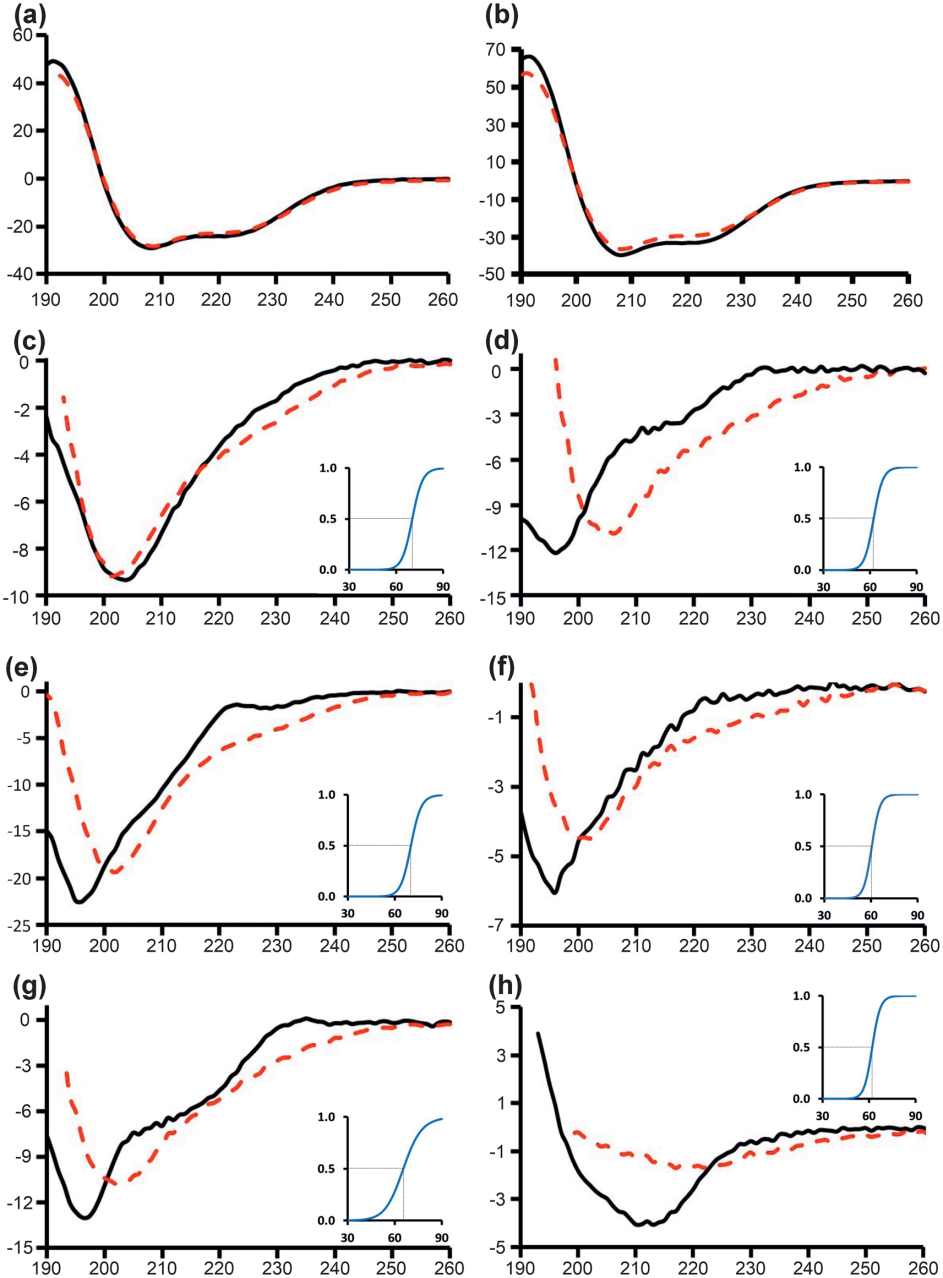
Far-ultraviolet circular dichroism (CD) spectra for the purified fibulin-2 constructs. The spectra are: (**a**) wild-type S427-G545, (**b**) S427-G545 (C500L), (**c**) G592-Q710, (**d**) D709-V802, (**e**) T800-V896, (**f**) V894-V981, (**g**) E979-L1063, and (**h**) K1061-P1221. The y-axis corresponds to mean residue ellipticity (deg.cm^2^dmol^−1^ × 10^3^) and x-axis is wavelength (nm). The CD spectra at room temperature (black solid line) and an average of high temperatures scans between 80–90 °C (red dash line). The insert panel shows the proportion of the higher temperature state as a function of temperature.

The secondary structure of the protein constructs was examined using far‐ultraviolet circular dichroism (CD) spectroscopy. The construct S427-G545, containing only the anaphylatoxin-like modules, is predicted to be predominantly α-helical (supplementary Fig. S1b). The CD spectra for S427-G545 for both wild-type and the C500L mutant was consistent with the predicted structural information, showing a positive peak near 193 nm and negative peaks near 208 nm and 222 nm (Fig. 4a,b). The CD spectral data for most of the fibulin-2 constructs containing two EGF-like domains, (D709-V802, T800-V896, V894-V981 and E979-L1063) expect G592-Q710, showed a sharp negative peak near 195 nm. This indicates that these constructs lack significant regular secondary structure components (Fig. 4c–g) which agrees with the AlphaFold predicted structures (supplementary Fig. S1c). In contrast, the CD spectra for construct K1061-P1221, which has a single EGF-like domain and Domain III, showed a positive peak near 190–195 nm and a negative peak near 210–220 nm (Fig. 4h) which suggests the presence of significant amounts of antiparallel β-pleated sheets. This agrees with the predicted structure of the C-terminal Domain III (supplementary Fig. S1d). Hence all fibulin-2 constructs exhibited CD spectra consistent with their predicted structure, which, when combined with the MS data that showed all cysteines are in disulfides, suggests that all are natively folded.

To further investigate the thermal stability of these constructs, changes in secondary structural elements were examined using CD spectrometry. The anaphylatoxin-like modules containing construct S427-G545, wild-type and C500L mutant, showed no or very minor change in CD spectra (Fig. 4a,b) which indicated that they are highly thermostable. In contrast most of the EGF-like domain containing constructs showed apparent conformational changes with a shift in the position of the negative peak to higher wavelengths at higher temperatures. The transition temperature was above 60 ˚C for most of the constructs and showed a single thermal transitional shift (Fig. 4c–g). A more significant change in CD spectra can be seen for K1061-P1221 between room temperature and high temperature, which is consistent with the thermofluor data (Fig. 3 and Fig. 4h). When the pre-heated samples (at 90 ˚C) were cooled to room temperature, the CD spectra for all except K1061-P1221 constructs shifted back to their native room-temperature state (supplementary Fig. S3). This efficient “refolding” could either be due to this being a conformational change rather than denaturation (consistent with the thermofluor data) or could be due to the presence of multiple disulfide bonds allowing the denatured protein to rapidly and efficiently readopt its native state.

### Structural studies

**Figure 5:**
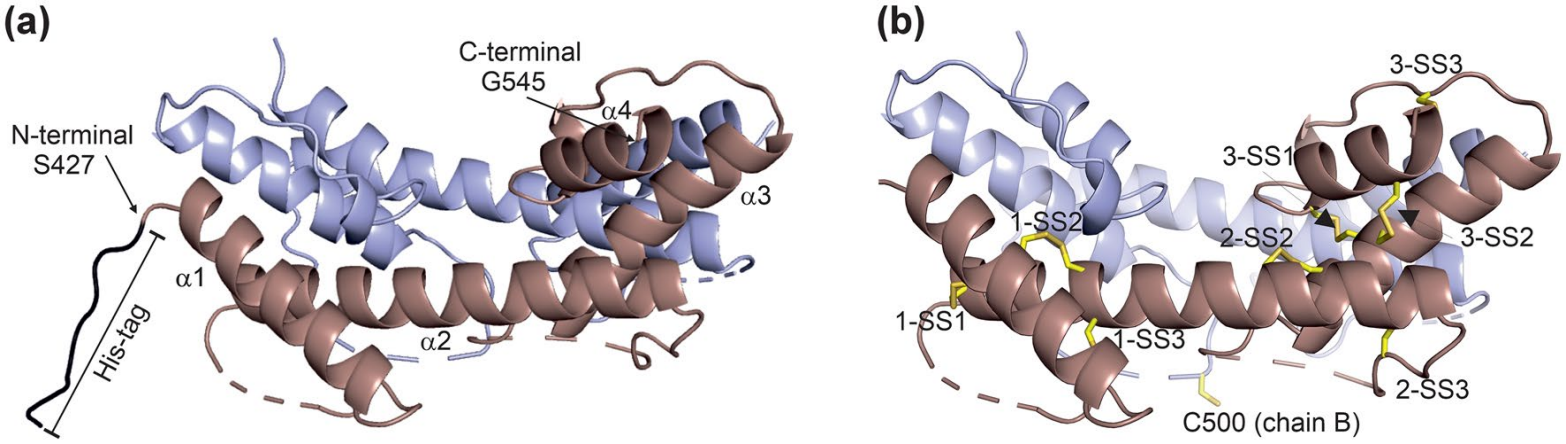
Crystal structure of mouse fibulin-2 construct S427-G545. This construct consists of three anaphylatoxin modules arranged in a tandem repeat fashion. (**a**) A cartoon representation of the structure, where chain A is represented in red, His-tag in black and chain B in blue. The N- and C-terminus of the construct are indicated. (**b**) The disulfide bond pattern of chain A of the S427-G545 dimer is shown as yellow stick representation. For disulfide nomenclature see the main text.

As no crystal structures were previously reported for any fibulin, we then attempted to crystallize the S427-G545 construct. We were able to obtain diffracting crystals and solved the structure in the P2_1_ space group at 2.2 Å resolution. The final model included 8 protein copies (A-H) and 108 water molecules in the asymmetric unit. A pseudo-translational symmetry was detected in the crystal form leading to the relatively high R factors at the end of the refinement; these being 24.28% (R_work_) and 29.74% (R_free_) (supplementary Table S1). However, the electron density maps were well defined for most of the protein chains (supplementary Fig. S4a). The structure included four dimers (AB, CD, EF and GH) in the asymmetric unit, with each dimer in a local two-fold symmetry. Pseudo-translational NCS symmetry was found between dimers AB, CD and EF, GH, respectively. The Cα trace of all the 8 copies in the asymmetric unit were very similar and superimposed with each other with r.m.s.d. values less than 1 Å. The overall structure of each chain is predominantly α-helical, which is consistent with the CD data (Fig. 4a). There are four alpha helices (T428-D445 for α1, D460-E488 for α2, L504-A520 for α3, Y533-E544 for α4) which all run in the same direction (Fig. 5). The loop structures between α1 and α2 (N446-S459) and between α2 and α3 (G499-S503) were incompletely modelled in all 8 chains indicating the flexible nature of these regions. The complete N-terminal His-tag with the initiating methionine was visible in chains A and E. The r.m.s.d. value for Cα atoms and for all atoms were 1.7 and 2.9, respectively, when chain A of the crystal structure was compared with the corresponding region of the AlphaFold2 (alphafold.ebi.ac.uk) model of mouse fibulin-2.

**Figure 6:**
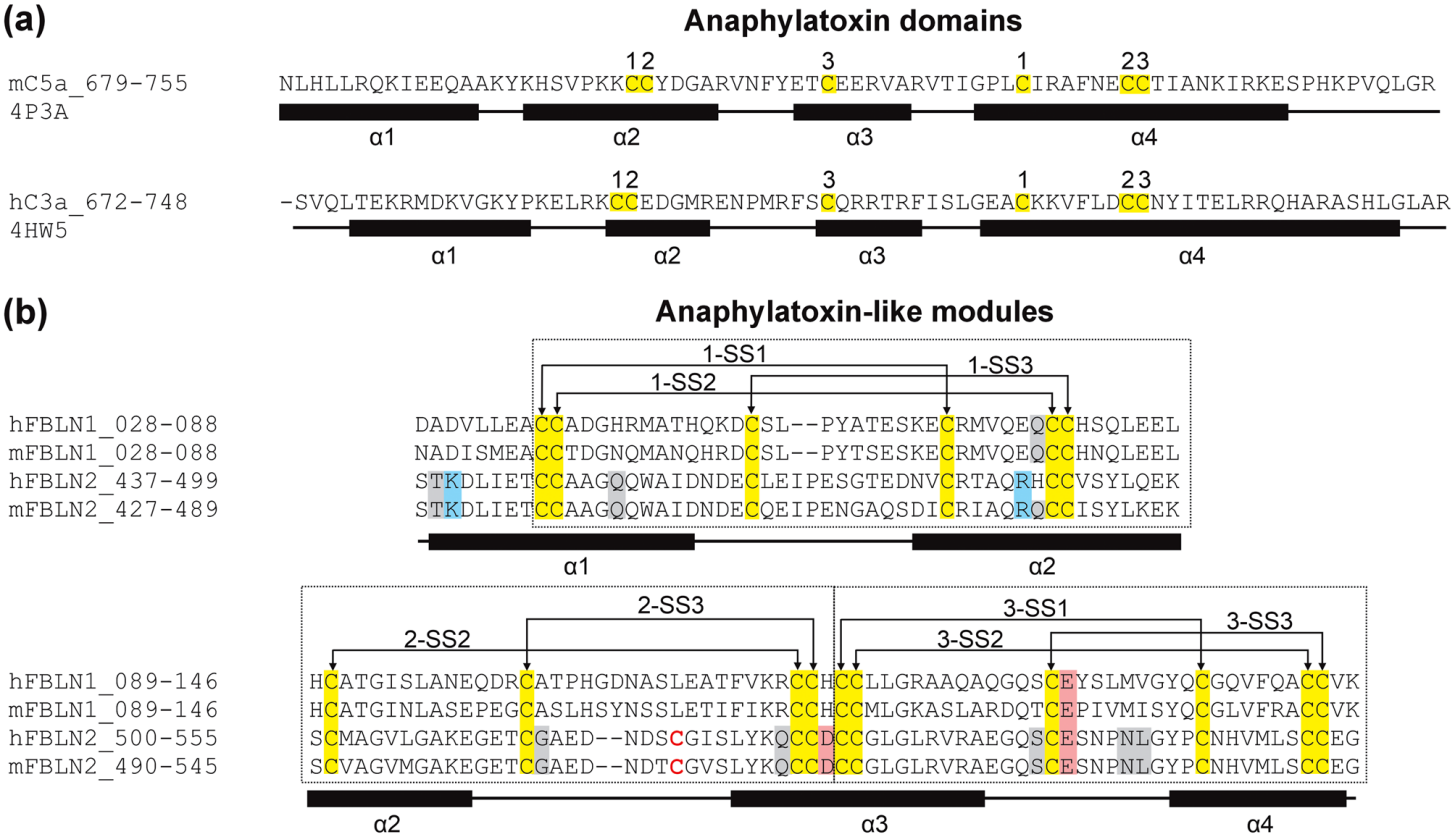
Comparison of the single anaphylatoxin domains and the three anaphylatoxin-like modules found in fibulins. (**a**) The primary and secondary structures of the anaphylatoxin domains of C3a (PDB code 4HW5) ^29^ and C5a (PDB code 4P3A) ^30^. The domain consists of four α-helices, the three stabilizing disulfides bridging the α2-α4 helices. (**b**) Sequence alignment of the S427-G545 region of human and mouse fibulins 1 and 2 including three anaphylatoxin-like sequence motifs. The secondary structure (α-helix) positioning is based on the current crystal structure of mouse fibulin-2. The numbering of cysteine residue represents the disulfide bonding partners. The unpaired C500 which forms an inter-molecular disulfide homodimer is shown as red. All the cysteines which are conserved are highlighted in yellow. The residues involved in non-covalent interactions in the dimer are highlighted in grey (hydrogen bonding) and in red/blue (electrostatic interactions).

The crystal structure shows a compact one-domain alpha helical structure rather than the previously suggested three separate domains each having anaphylatoxin-like motif^4^. The structure has a disulfide bond architecture which stabilizes the three anaphylatoxin modules (Fig. 5b and Fig. 6b). The first anaphylatoxin-like fold includes the α1-helix (including C435 and C436), α1-α2 loop (including C449), and the N-terminal half of the long α2-helix (including C462, C469 and C470) and the fold is stabilized by three disulfides C435-C462, C436-C469 and C449-C470. These disulfide bonds are referred to as 1-SS1, 1-SS2 and 1-SS3, respectively, from now on. The second anaphylatoxin-like fold includes the C-terminal half of α2-helix (including C479), α2-α3 loop (including C492) and N-terminal half of α3-helix (including C508 and C509). This fold is stabilized by two disulfides (C479-C508 and C492-C509 referred to as 2-SS2 and 2-SS3, respectively). The third anaphylatoxin-like fold consists of the C-terminal half of the α3-helix (including C511 and C512), α3-α4 loop (including C525) and the complete α4-helix (including C535, C542 and C543), and is again stabilized by three disulfides namely C511-C535 (3-SS1), C512-C542 (3-SS2) and C525-C543 (3-SS3). In total, 8 intra-molecular disulfide bonds are found in the domain. Most of them are clearly defined by the electron density (such as 1-SS3 (C436-C469) shown in supplementary Fig. S4a) and they have the same conformation in all 8 copies of the protein in the asymmetric unit.

Structural alignment analysis using the DALI sever^28^ does not show any homologous structure with a significant Z-score value. A structural comparison between the solved crystal structure with previously solved structures of wild-type anaphylatoxin domain, human C3a (PDB code 4HW5)^29^ and murine C5a (PDB code 4P3A)^30^, was done. These structures have a similar fold, having four α-helices and three conserved disulfide bonds (Fig. 6a and Fig. S5a) which help in stabilizing the protein. These crystal structures, 4HW5 and 4P3A, also have similar disulfide bond arrangement having the 1st, 2nd cysteine residues located on α2-helix, the 3^rd^ cysteine on α3-helix and the cysteine residues which form disulfide bonds with them being all located on α4-helix, as shown in Fig. 6a and Fig. S5. In contrast, the fibulin-2 anaphylatoxin-like modules lack the α1-helix which is found in the anaphylatoxin domain structures (Fig. 6a). Additionally, two α-helices, namely α2 and α3, in fibulin-2 are part of two different anaphylatoxin modules (Fig. 6b). All of this suggests that in fibulin-2, the three anaphylatoxin modules are all embedded into a single-domain four-helix structure forming a dimer with covalent bonding via C500.

The inter-molecular disulfide bond between the two chains between C500 cannot be confirmed reliably with this crystal structure. Cys500 locates in the flexible α2-α3 loop, which is only partly visible in the electron density maps. In the current crystal structure, Cys500 has been modelled in chains B and F. In these chains, electron density is clear for C500-L504 of the α2-α3 loop. However, the N-terminal region of this loop, A494-T499, in those two chains has only weak density and it was not possible to reliably build this region (supplementary Fig. S4b). However, the (weak) electron density near C500 in chain F suggests the presence of an intermolecular disulfide with C500 of chain E (supplementary Fig. S4b). When combined with the non-reducing SDS-PAGE (Fig. 2b) and MS (Table 2), which both indicate that all of the protein is in a disulfide linked homodimer, the crystal structure data implies that the inter-subunit disulfide is formed, but that it is not stabilizing the flexible nature of the α2-α3 loop.

To examine potential dimerization of the full-length protein, we calculated dimeric models of the complete fibulin-2 molecule using AlphaFold multimer^31^. This modelling resulted in five different proposed dimeric structures. The one with the highest probability score is shown supplementary Fig. S6. Interestingly, all five models show a common dimerization formation with anaphylatoxin-like modules at the interface between monomers. Furthermore, the formed anaphylatoxin-like region dimer is very similar with the crystal structure presented in this study. This further suggests that the anaphylatoxin-like module region is a key player in the dimerization of the fibulin-2 protein. Interestingly, none of the models showed head-to-tail dimerization as predicted previously^10^.

## Methods

### Cloning, expression, and purification

The use of bioinformatics tools for defining construct boundaries and plasmid design as well as the construction of plasmids was performed as previously described^25^. Briefly, all the plasmids used in this study (shown in Table 1), have an N‐terminal hexa-histidine (MH_6_M‐) tag preceding the first amino acid of the protein sequence. To confirm that the disulfide bond was not essential for dimer formation we made the cysteine 500 to leucine (C500L) mutation in the S427-G545 construct. The construction of mutant was made using the QuikChange site-directed mutagenesis kit (Agilent) according to the manufacturer’s instructions. All genes were fully sequenced prior to expression.

The plasmid containing the gene of interest (Table 1) along with the CyDisCo plasmid (pMJS205) containing, Erv1p and PDI^32^, were co-transformed in BL21 (DE3) (from Stratagene) *E. coli* strain and grown in terrific broth autoinduction media (Formedium) at 30 ºC prior to induction and at 15 °C during the induction phase. Bacterial cell pellets from 1 L cultures were re-suspended in 400 mL of lysis buffer (20 mM Tris–HCl pH 8, 150 mM NaCl, 2 mM CaCl_2_, 15 mM imidazole and 20 µg/mL DNase). The cells were lysed by sonication for a total duration of 90 s with 5 s pulse on and 25 s pulse off at 40% amplitude. The lysate was centrifuged at 30,000 × g, 4 °C for 40 min, the supernatant collected and filtered through a 0.45 µm filter.

The first step of purification used a HiTrap™ 5 mL chelating HP column (GE Healthcare) for nickel immobilized metal affinity chromatography (IMAC), where 1 column volume (CV) is equal to 5 mL. The column was first washed with 5 CV of water followed by 1 CV of nickel chloride. Excess unbound nickel was removed by washing the column with 5 CV of water. The column was then equilibrated with 5 CV equilibration buffer containing 20 mM Tris–HCl pH 8, 150 mM NaCl, 2 mM CaCl_2_. The soluble filtered supernatant fraction was then loaded onto the column followed by a 3 CV equilibration buffer. The column was washed using 10 CV of 20 mM Tris–HCl pH 8, 150 mM NaCl, 2 mM CaCl_2_, 50 mM imidazole. The protein of interest was eluted by applying a linear gradient of 20 mM Tris–HCl pH 8, 150 mM NaCl, 2 mM CaCl_2_, 300 mM imidazole over 10 CV. The IMAC elution fractions containing the target protein were pooled and concentrated. Approximately 1 mL of the protein was then injected into the HiLoad™ 16/600 Superdex™ 75 pg column (GE Healthcare) for size exclusion chromatography (SEC) purification. The column was pre- equilibrated with 20 mM Tris–HCl pH 8, 150 mM NaCl, 2 mM CaCl_2_. Calcium chloride was absent during the purification for the S427-G545 constructs.

### Biophysical characterization

The characterization of purified protein samples by sodium dodecyl sulfate- polyacrylamide gel electrophoresis (SDS-PAGE), mass spectrometry (MS), far‐ultraviolet circular dichroism (CD) and thermofluor assay, were followed as described previously^25^. The MS analysis shown here was done using the NEM treated sample. It showed the same results as native samples. Additionally, an addition of 57 Da was observed for all constructs which is most likely is due to the presence of bound nickel from the IMAC purification. Size exclusion chromatography coupled with multi angle light scattering (SEC-MALS) was performed to determine the oligomeric state of the wild-type and mutant of S427-G545 construct. The analysis was carried out using a Wyatt Mini DAWN instrument connected to a Shimadzu HPLC system. The SEC purified protein sample (50 µL of ~ 2 mg/mL) was injected into the pre-equilibrated superdex 200 Increase 10/300 column with 20 mM Tris–HCl pH 8, 150 mM NaCl. The flow rate was kept at 0.5 mL/min and the analysis of MALS data was done using ASTRA software version 7.0 (Wyatt technologies).

### Structural characterization

Structural studies of the pAS79 construct (MH_6_M-S427-G545) having a protein concentration of 10 mg/mL was carried out using x-ray crystallography. Initially crystallization screening was set up on 96-well triple sitting drop plate (sptlabtech) using a Mosquito LCP nanodispenser (sptlabtech). The crystallization screens used included Oulu Factorial (in house screen) and JCSG plus (Molecular Dimensions). The volume of reservoir solution (RS) used was 50 µL and the crystallization drops composition was in 1:2, 1:1 and 2:1 ratio for protein:RS. The crystallization plates were kept at room temperature (22 °C) and formulatrix rock imagers RI54 was used for plate imaging. Crystallization drops were viewed using IceBear software^33^.

An initial crystallization hit was found using 0.2 M potassium nitrate, 20% w/v PEG 3350. The condition was further optimized by using different protein concentrations ranging from 2.5—10.0 mg/mL and varying the PEG 3350 concentration from 2—25%. A single crystal appeared in a crystallization drop with a 1:2 (protein:RS) ratio composed of 0.2 M potassium nitrate, 2% w/v PEG 3350 with a stock concentration of protein of 10 mg/mL. The protein crystal was mounted in a cryoloop (Hampton) with the addition of 25% glycerol in the reservoir solution prior to flash freezing in liquid nitrogen. The x-ray diffraction data from the frozen crystal was collected to 1.97 Å resolution at Diamond Light Source (DLS, Didcot, United Kingdom) beamline I-04. The auto-processed data (48.40–1.97 Å) from Xia2 (3dii) pipeline was used for structure solution^34^. The data collection parameters and structure solution parameters are shown in supplementary Table S1.

The anaphylatoxin-like region (S427-G545) of the AlphaFold predicted model of the complete fibulin-2 from mouse (accession code: AF-P37889-F1, https://alphafold.ebi.ac.uk)^26,27^, was used as a search model in the molecular replacement calculations by Phaser of the Phenix suite^35,36^. Initial refinement steps were done with Phenix.refine^37^ and the iterative model building by using COOT^38^ by using the data up to 2.2 Å resolution. The diffraction data in the resolution range 2.19–1.97 Å were not used for the refinement as the reflections were very weak (I/sigma below 1.8, R_merge_ and R_pim_ more than 100% and 70% respectively) and their inclusion did not bring any improvements to the electron density maps. The last refinement was done with Refmac^39^ in the CCP4 Cloud^40^. The final coordinates were validated with MolProbity^41^. Images of the structures were prepared using Pymol software^42^.

### Modelling calculations

The dimeric models for the complete full-length mouse fibulin-2 were calculated by AlphaFold-multimer^31^ in the COSMIC^2^ cloud platform for structural biology research and education^43^.

### Supplementary Information

Supplementary Information.

## Data Availability

The data presented in this study is contained within the article and supplementary material. The structure is available in (PDB code: 8R5W).
