# Supplementary material for: Biophysical and structural studies of fibulin-2
Source: Sci Rep. 2024 Jul 2;14:15091. doi: 10.1038/s41598-024-64931-7 (PMC11220139; doi:10.1038/s41598-024-64931-7)
Supplement: Supplementary file 1 — Supplementary Information. [file 41598_2024_64931_MOESM1_ESM.pdf]

Supplementary information

Biophysical and structural studies of fibulin-2 fragments

Anil A. Sohail, M. Kristian Koski, and Lloyd W. Ruddock

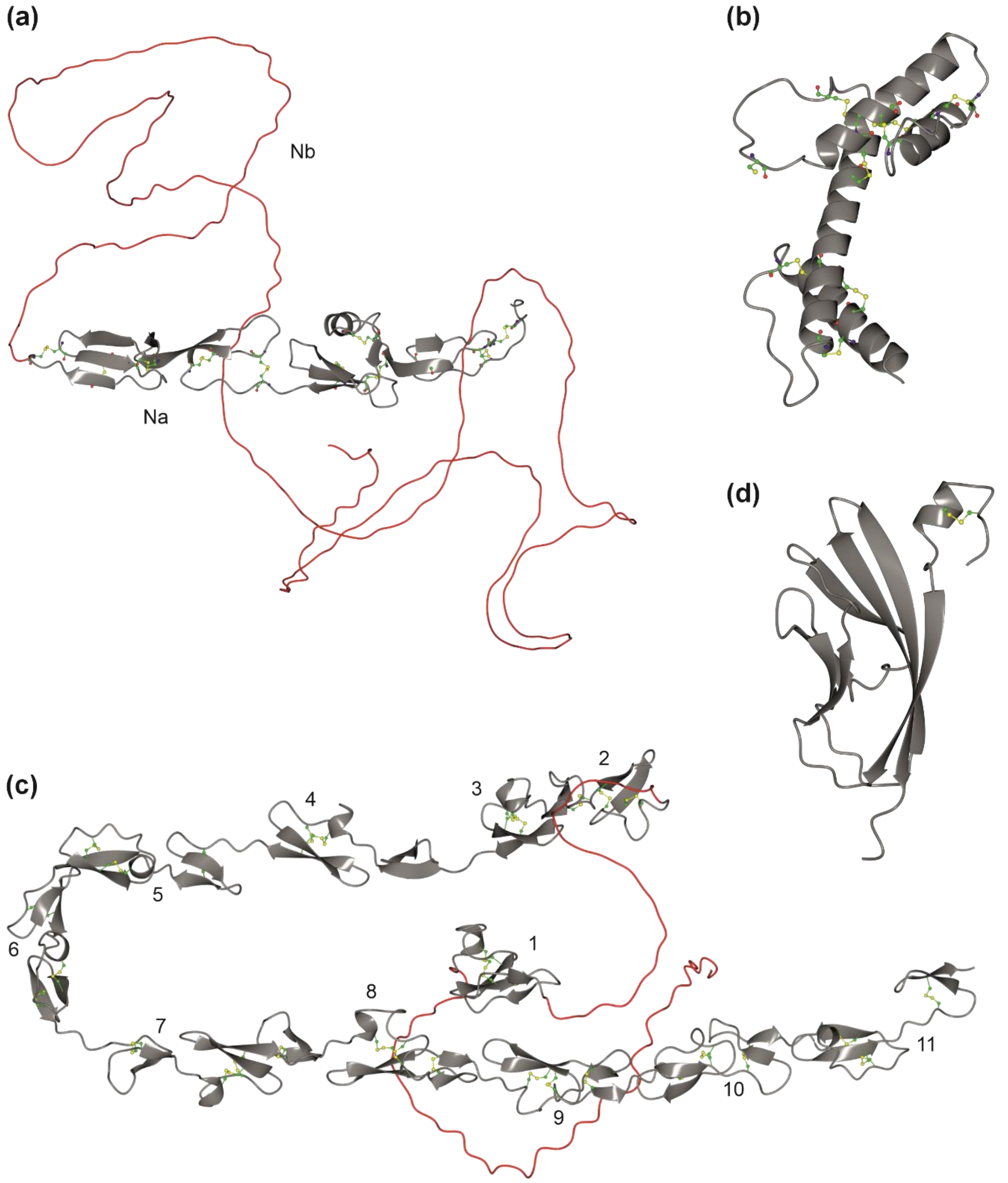

Figure S1. AlphaFold predicted structures of fibulin-2. Ribbon representations of the domains extracted from the AlphaFold predicted monomeric model of full-length mouse fibulin-2 protein (accession code: AF-P37889-F1). **a)** N-terminal part of region 1 which is composed of the cysteine rich N_a domain (grey) and the cysteine lacking N_b domain (predicted to be fully unstructured, red). **b)** The C-terminal part of region I containing the three anaphylatoxin-like motifs (grey). The predicted structure is very similar and the disulfide bond architecture the same as in the crystal structure presented in this study. **c)** Region 2 containing the 11 EGF-like domains (grey) along with two linker regions with no predicted regular secondary structure (red). **d)** The Domain III of region 3 predicted to consist of mainly β-sheet structure (grey). All the secondary structure predications have a high confidence level (above 90 %). Disulfide bonds (yellow) are shown as ball and stick representation.

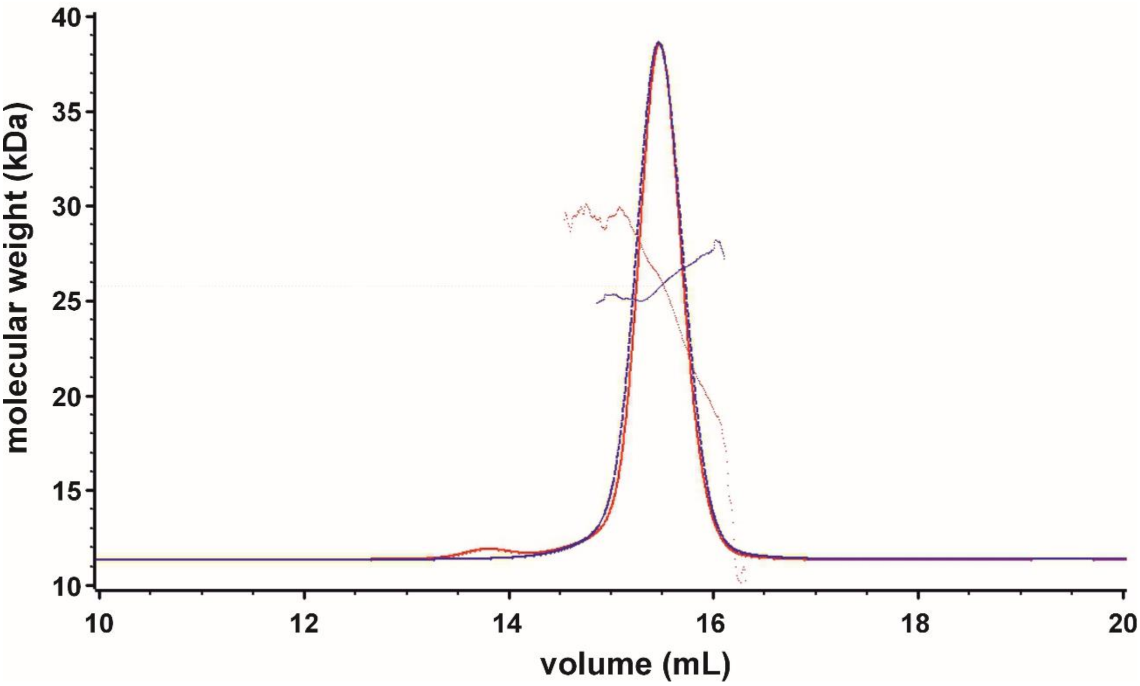

Figure S2. SEC-MALS analysis of the S427-G545 fragment of mouse fibulin 2. The light scattering signals of the wild type (red) and C500L mutant (blue) are shown. In addition, the molecular mass distribution profiles of both runs are shown for the peak regions (horizontal lines). The molar masses (~26.3 kDa for the wild type and ~25.7 kDa for the C500L mutant) were calculated from the peak fractions and using the refractive index (RI) and light scattering signals with Astra software (Wyatt Technologies).

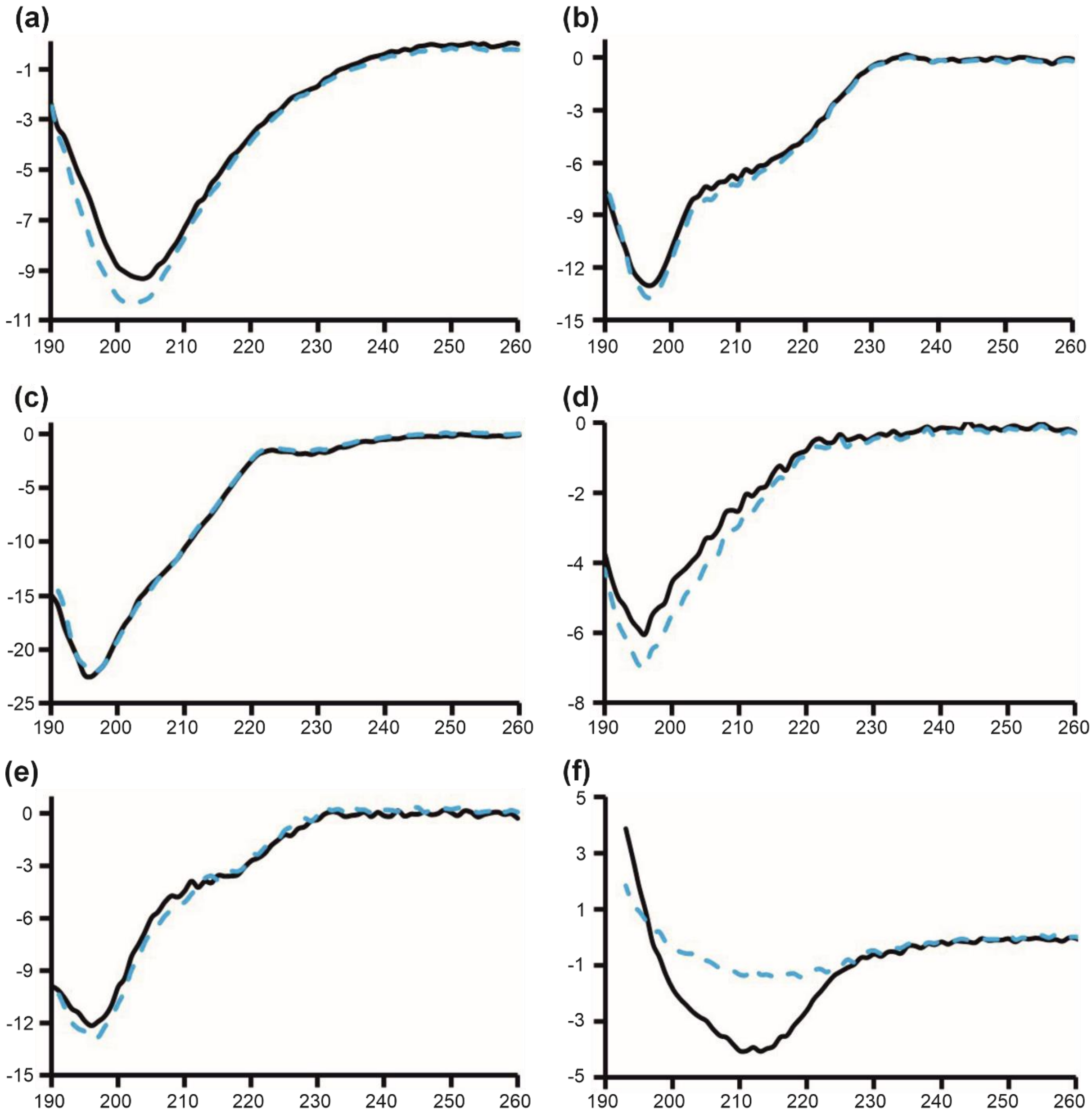

Figure S3. Far-ultraviolet circular dichroism (CD) spectra for the purified fibulin-2 fragments. The spectra are: **a)** G592-Q710, **b)** D709-V802, **c)** T800-V896, **d)** V894-V981, **e)** E979-L1063, and **f)** K1061-P1221. The y-axis corresponds to mean residue ellipticity (deg.cm^{2}dmol^{-1}) x10^3 and the x-axis is wavelength (nm). The CD spectra at room temperature (black solid line) and CD spectra for refolded sample (blue dash line) which were pre-heated to 90 °C then cooled to room temperature. All constructs except K1061-P1221 show similar CD spectra after the heating-cooling cycle.

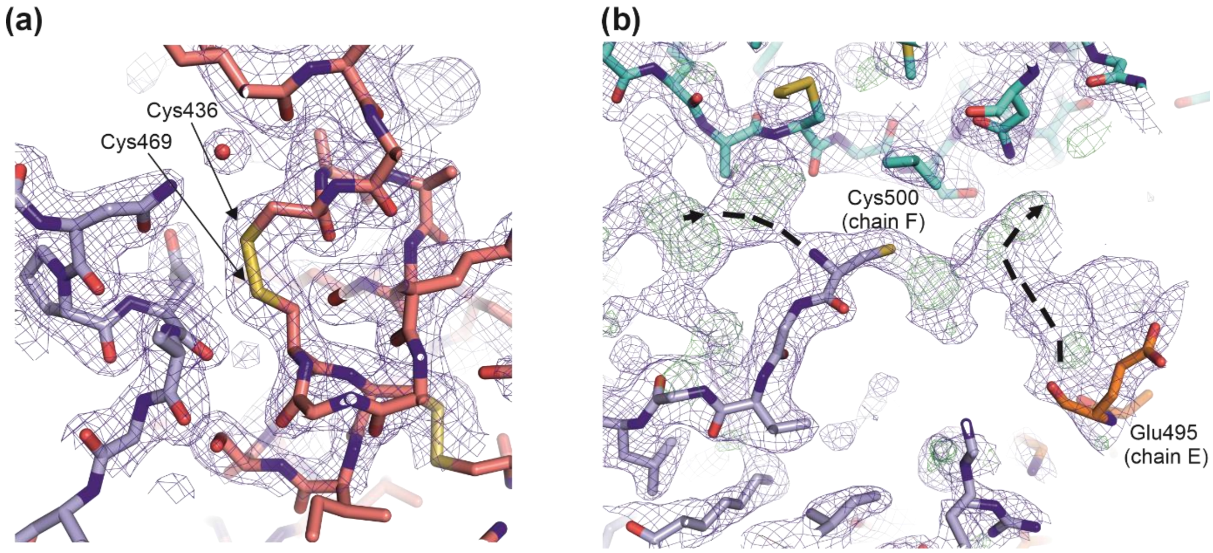

Figure S4. Electron density map of the crystal structure of the S427-G545 fragment of mouse fibulin 2. a) The 2Fo-Fc map around the C436-C469 disulfide (1-SS2) bond in chain A contoured at 1.0 σ. **b)** The 2Fo-Fc map (blue, countered at 0.6 σ) and the Fo-Fc map (positive density, green, countered at +2.0 σ) around C500 of chain F. The loop region between α2 and α3 including C500 is flexible in all chains of this crystal structure. In chain F, the region just before the C500 (E494-T499) has some continuous electron density at low sigma levels (shown with dashed line before C500(F)), but the quality was not good enough for reliable model building. The corresponding loop region in the chain E (the dimer partner of chain F), has also a very weak electron density shown only at low sigma levels (the missing region, D496-V502, shown with a dashed line). However, the electron densities do support the concept of a disulfide bond between C500(F) and C500(E).

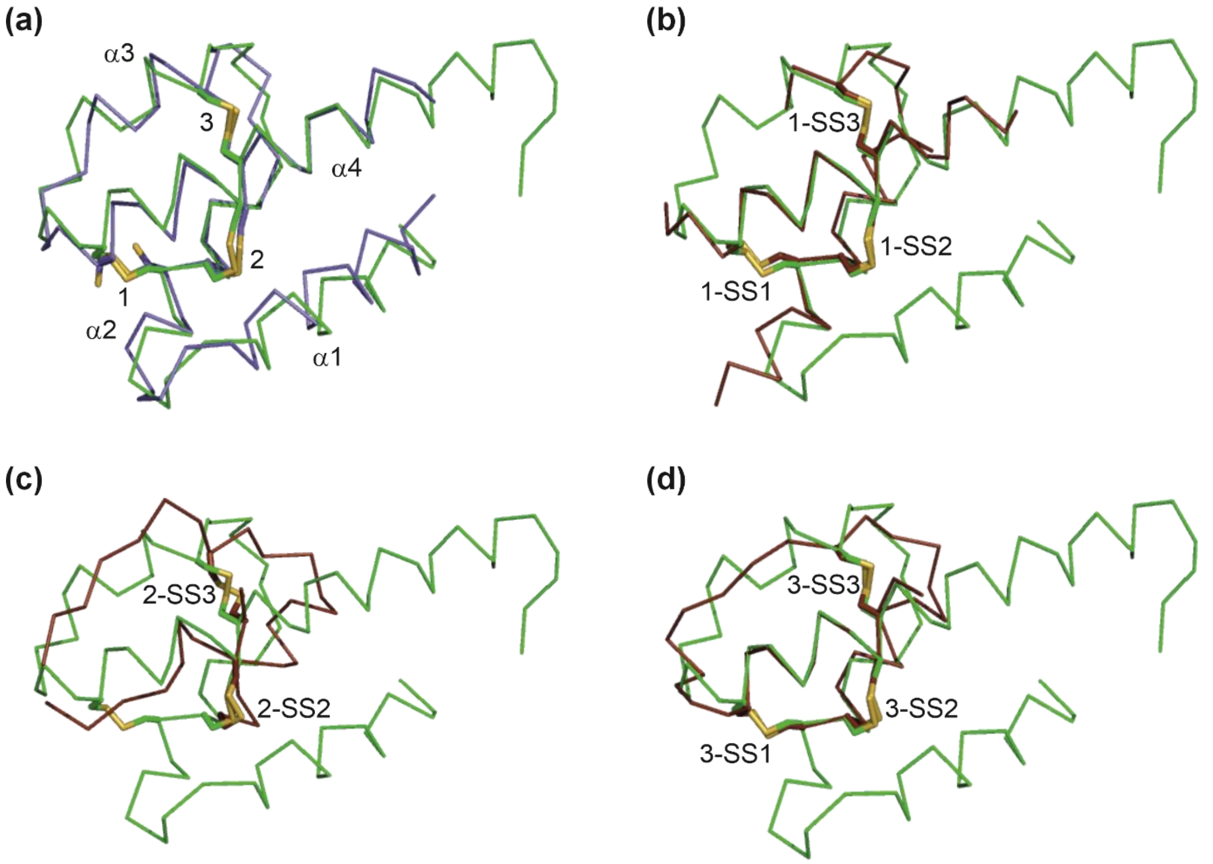

Figure S5. Structural alignment analysis. Ribbon representations of anaphylatoxin structures with C3a anaphylatoxin domain (PDB code 4HW5) [29] in green as a reference structure. **a)** Superimposed with C5a (PDB code 4P3A) [30] in blue, with a r.m.s.d. value of 1.408 Å. **b)** Superimposed with module 1 of solved structure having an r.m.s.d. value of 1.172 Å. **c)** Superimposed with module 2 of solved structure having r.m.s.d. of 5.476 Å. **d)** Superimposed structure with module 3, having a r.m.s.d. value of 0.727 Å. Disulfide bonds are shown as yellow stick representations.

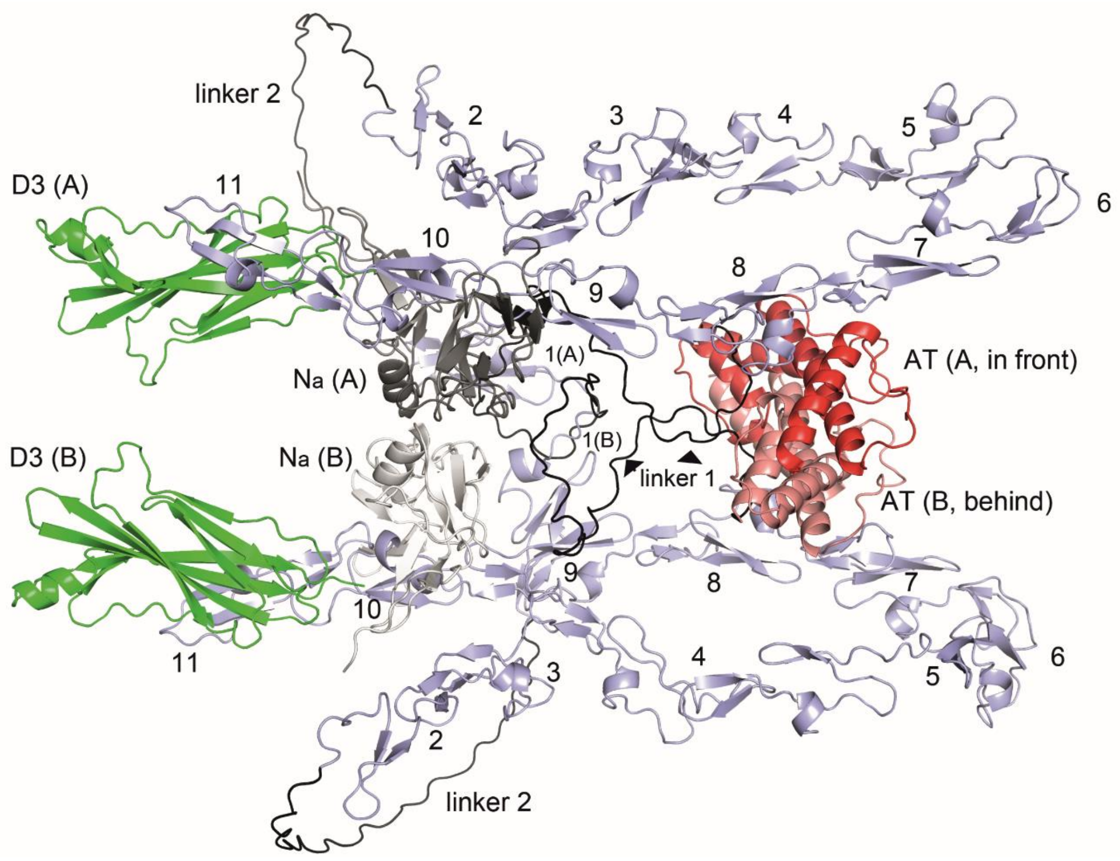

Figure S6. Homodimer model of mouse fibulin-2. A cartoon representation of one possible homodimer model of the full-length mouse fibulin-2 protein as calculated by AlphaFold multimer. Chain A and chain B of the N_a and anaphylatoxin-like (AT) domains are shown in dark and light grey and red, respectively. The EGF-like domains are shown in blue and the two, unassigned sequence regions, linker 1 (between AT and EGF-like domain I) and linker 2 (between the first and the second EGF-like domains) are shown in black. The C-terminal domain III is shown in green. The long and fully unstructured domain N_b is not shown for both chains. The coloring scheme is the same as in **Figure 1**. In this model, the key dimeric interactions are made between the Na and anaphylatoxin regions, as well as the linker 1 region. The anaphylatoxin dimer of the full-length fibulin-2 model shown here is highly similar to the experimentally determined crystal structure of the anaphylatoxin dimer described in this paper.

Table S1: Data collection and statistics of the data processing and refinement of the structure of the S427-G545 fragment of mouse fibulin-2

**Data collection**	
Beam line	I04 (DLS, UK)
Wavelength	0.954
Detector	EIGER2 XE 16M
Temperature (K)	100
Data processing software	XDS/Aimless
**Data processing statistics**	
Unit cell parameters (Å,°)	a = 103.68, b = 34.51, c = 106.89<br>α = 90.00, β = 94.00, γ = 90.00
Space group	$P2_1$
Resolution range (Å)[1]	48.80-1.97 (2.02-1.97)
V_m ($Å^3$/Da)	1.73
Molecules per asymmetric unit	8 (4 homodimers)
Number of observations (total)	369903 (28372)
Number of observations (unique)	54442 (4023)
Multiplicity	6.8 (7.1)
Completeness (%)	99.9 (99.9)
R_{merge} (%)	10.9 (316)
R_{pim} (%)	4.5 (127)
I/σ(I)	8.4 (0.7)
CC(1/2)	0.999 (0.366)
**Refinement statistics**	
Resolution (Å)	48.80-2.20
R_{work} (%)	24.28
R_{free} (%)	29.74
Number of unique reflections	39272
Number of atoms[2]	6605
Average B-factors ($Å^2$)	58.34
Rmsd bond length (Å)	0.0076
Rmsd bond angle (°)	1.5320
Ramachandran plot	
Favored (%)	98.3
Allowed (%)	1.7
Outliers (%)	0
**PDB entry**	**8R5W**

1. The numbers in parentheses refer to the outer shell.
2. Non-hydrogen atoms.
